# A183 INVESTIGATING THE ROLE OF HUMAN BETARETROVIRUS (HBRV) INFECTION IN THE PATHOGENESIS OF CROHN’S DISEASE AND ULCERATIVE COLITIS

**DOI:** 10.1093/jcag/gwae059.183

**Published:** 2025-02-10

**Authors:** D Waly, H Syed, O Oyegbami, N Sun, N Hotte, E Lerner, H Armstrong, E Wine, K Madsen, T Perry, A Mason

**Affiliations:** medicine, University of Alberta, Edmonton, AB, Canada; medicine, University of Alberta, Edmonton, AB, Canada; medicine, University of Alberta, Edmonton, AB, Canada; medicine, University of Alberta, Edmonton, AB, Canada; medicine, University of Alberta, Edmonton, AB, Canada; medicine, University of Alberta, Edmonton, AB, Canada; medicine, University of Alberta, Edmonton, AB, Canada; medicine, University of Alberta, Edmonton, AB, Canada; medicine, University of Alberta, Edmonton, AB, Canada; medicine, University of Alberta, Edmonton, AB, Canada; medicine, University of Alberta, Edmonton, AB, Canada

## Abstract

**Background:**

We have been investigating the role of viral infections in the development of inflammatory bowel disease (IBD) in genetically predisposed individuals. Using the SvEv IL10 -/- model of colitis, we found evidence of mouse mammary tumor virus (MMTV) infection and modulated the disease with combination antiretroviral therapy. Reducing MMTV RNA in the colon lowered inflammation, cytokine levels, and microbial dysbiosis. Our results showed viral superantigen (SAg) activity, which MMTV uses to replicate in lymphocytes. MMTV also exploits IL-10 to tolerize the host, and we hypothesize that IL-10 deficiency leads to colitis in this model (Armstrong et al., Microbiome, 2023).

**Aims:**

This study investigates SAg activity in patients with IBD, focusing on Crohn’s disease (CD) and ulcerative colitis (UC) by analyzing T cell receptor variable beta region (TCR-Vβ) skewing. Additionally, we assess TCR diversity and selective enrichment of specific TCR-Vβ families by comparing IBD patients with healthy subjects and analyzing TCR profiles between active and inactive disease.

**Methods:**

Peripheral blood samples (n > 2500) from the IBD Plexus database were analyzed to evaluate TCR-Vβ skewing as an indicator of SAg activity in patients with IBD. Comparisons of TCR-Vβ family usage were made between healthy subjects and those with CD and UC. Statistical analyses were performed in R, using unpaired t-tests and Bonferroni correction. TCR diversity was assessed using the Inverse Simpson Index (ISI) for CD, UC, and age-matched healthy controls. ISI values were also compared between active and inactive disease states.

**Results:**

An analysis of TCR repertoires from PBMCs in CD (n=1901), UC (n=913), and healthy controls (n=725) revealed greater SAg activity in CD and UC patients than in controls. In healthy individuals, the TCR-Vβ repertoire was diverse and polyclonal, while IBD patients had skewed repertoires. TRBV-03, TRBV-09, and TRBV-24 were consistently enriched in CD and UC patients across all age groups. The inverse Simpson’s index showed increased T cell clonality in CD and UC patients. Additionally, TCR diversity was significantly lower in patients with active disease than in those with inactive disease.

**Conclusions:**

Our study underscores the role of SAg activity in IBD, particularly in CD and UC. We observed notable TCR-Vβ skewing and TRBV subset enrichment in IBD patients, accompanied by increased T cell clonality, especially in active disease. This selective skewing of TCR-Vβ subsets suggests involvement of distinct SAgs. While bacterial SAgs are often linked to severe disease, our results indicate a contribution from non-bacterial SAgs, highlighting a potential connection between SAg activity and the onset of IBD.

**Funding Agencies:**

CCC

